# Safety, Tolerability, and Immunogenicity of Interferons

**DOI:** 10.3390/ph3041162

**Published:** 2010-04-20

**Authors:** Michael G. Tovey, Christophe Lallemand

**Affiliations:** Laboratory of Viral Oncology, FRE2937 CNRS, Institut André Lwoff, 7 rue Guy-Moquet, 94801 Villejuif, France

**Keywords:** cytokines, interferons, interleukins, innate immunity, Toll-like receptors

## Abstract

Interferons (IFNs) are class II cytokines that are key components of the innate immune response to virus infection. Three IFN sub-families, type I, II, and III IFNs have been identified in man, Recombinant analogues of type I IFNs, in particular IFNα2 and IFNβ1, have found wide application for the treatment of chronic viral hepatitis and remitting relapsing multiple sclerosis respectively. Type II IFN, or IFN gamma, is used principally for the treatment of chronic granulomatous disease, while the recently discovered type III IFNs, also known as IFN lambda or IL-28/29, are currently being evaluated for the treatment of chronic viral hepatitis. IFNs are in general well tolerated and the most common adverse events observed with IFNα or IFNβ therapy are “flu-like” symptoms such as fever, headache, chills, and myalgia. Prolonged treatment is associated with more serious adverse events including leucopenia, thrombocytopenia, increased hepatic transaminases, and neuropsychiatric effects. Type I IFNs bind to high-affinity cell surface receptors, composed of two transmembrane polypeptides IFNAR1 and IFNAR2, resulting in activation of the Janus kinases Jak1 and Tyk2, phosphorylation and activation of the latent cytoplasmic signal transducers and activators of transcription (STAT1) and STAT2, formation of a transcription complex together with IRF9, and activation of a specific set of genes that encode the effector molecules responsible for mediating the biological activities of type I IFNs. Systemic administration of type I IFN results in activation of IFN receptors present on essentially all types of nucleated cells, including neurons and hematopoietic stem cells, in addition to target cells. This may well explain the wide spectrum of IFN associated toxicities. Recent reports suggest that certain polymorphisms in type I IFN signaling molecules are associated with IFN-induced neutropenia and thrombocytopenia in patients with chronic hepatitis C. IFNγ binds to a cell-surface receptor composed of two transmembrane polypeptides IFGR1 and IFGR2 resulting in activation of the Janus kinases Jak1 and Jak2, phosphorylation of STAT1, formation of STAT1 homodimers, and activation of a specific set of genes that encode the effector molecules responsible for mediating its biological activity. In common with type I IFNs, IFNγ receptors are ubiquitous and a number of the genes activated by IFNγ are also activated by type I IFNs that may well account for a spectrum of toxicities similar to that associated with type I IFNs including “flu-like” symptoms, neutropenia, thrombocytopenia, and increased hepatic transaminases. Although type III IFNs share the major components of the signal transduction pathway and activate a similar set of IFN-stimulated genes (ISGs) as type I IFNs, distribution of the IFNλ receptor is restricted to certain cell types suggesting that IFNλ therapy may be associated with a reduced spectrum of toxicities relative to type I or type II IFNs. Repeated administration of recombinant IFNs can cause in a break in immune tolerance to self-antigens in some patients resulting in the production of neutralizing antibodies (NABs) to the recombinant protein homologue. Appearance of NABs is associated with reduced pharmacokinetics, pharmacodynamics, and a reduced clinical response. The lack of cross-neutralization of IFNβ by anti-IFNα NABs and *vice versa*, undoubtedly accounts for the apparent lack of toxicity associated with the presence of anti-IFN NABs with the exception of relatively mild infusion/injection reactions.

## 1. Introduction

Interferons (IFNs) are class II cytokines that play a determining role in the innate immune response to virus infection and in the establishment of the subsequent adaptive immune response leading to long-term antiviral immunity [1,2,3]. The human IFN super-family is comprised of three sub-families, type I, II, and III [3,4]. In man, the type I IFN family of intron-less genes is located on the short arm of chromosome 9 and encodes 12 functional IFNα, and single IFNβ, IFNω, IFNε, and IFNκ subtypes. All type I IFNs bind to a common high-affinity hetrodimeric cell surface receptor composed of two trans-membrane polypeptide chains, IFNAR1 and IFNAR2. IFN binding results in the phosphorylation and activation of two Janus kinases, Tyk2 and Jak1, associated with the cytoplasmic domains of IFNAR1 and IFNAR2 respectively, and the formation of docking sites for the latent cytoplasmic signal transducers and activators of transcription (STAT) 1 and 2. Tyk2 and Jak1, that in turn phosphorylate and activate STAT1 and STAT2, leading to the formation of a transcription complex in association with IFN regulatory factor 9 (IRF9). Translocation of this complex to the nucleus results in the transcriptional activation of a specific set of genes that encode the effector molecules responsible for mediating the biological activities of the type I IFNs including antiviral proteins such as MxA and 2’,5’ oliogadenylate synthetase, and immune modulatory proteins such as major histocompatibility antigens (MHC) [1,2,3,4]. Type I IFNs can, however, phosphorylate and activate other signaling molecules including STAT3, STAT4, Map kinases, and PI-3 kinase [5,6]. Although all the type I IFNs appear to induce comparable qualitative biological activities there are quantitative differences in the activity of different sub-types which is reflected in a different spectrum of clinical activity [3]. Several regulatory mechanisms exist for modulating the action of type I IFNs both at the level of receptor expression and at the level of subsequent down-stream signaling. Regulation of receptor expression is a common mechanism for modulating cytokine responsiveness and it has been shown that the type I IFN receptor is internalized and degraded after ligand binding [7,8,9], and that after prolonged IFNα therapy, patients displayed a state of decreased receptor expression, that correlated with a favorable response to IFN therapy [10,11]. In contrast, a negative correlation has been reported between expression of IFNAR2a, a soluble isoform of IFNAR2 produced by alternative splicing, and responsiveness to IFNβ treatment in patients with RRMS [11], suggesting that production of soluble IFNAR2a can sequester IFNβ thereby inhibiting IFNβ activity and constituting a negative feedback loop. A number of negative regulatory molecules have been described including SOCS-1, UBP43, PIAS, and SHP-2 that act to down-regulate IFN signaling in order to prevent over stimulation of the IFN signaling pathway that could lead to the development of autoimmunity. Thus, SOCS-1 binds to the C-terminal region of IFNAR1 preventing binding of Tyk2, while UBP43 blocks the interaction of Jak1 with IFNAR2. PIAS (protein inhibitor of activated STAT) promotes SUMOylation of STAT proteins including STAT3 in a manner that resembles the action of the RING-type ubiquitin E3 ligases. SHP-2 is involved in STAT dephosphorylation and regulation of nuclear export [12].

In man, a single type II IFN has been described commonly known as interferon gamma (IFNγ) which is encoded by a single copy intron-containing gene located on chromosome 12. IFNγ binds to a specific cell surface receptor composed of two trans-membrane polypeptides, IFNGR1 and IFNGR2 and signals through a STAT1 homo-dimer resulting in the transcriptional activation of a specific set of genes, that encode the effector molecules responsible for mediating its biological activity. A number of the genes activated by IFNγ are also induced by type I IFNs [3,4]. In contrast to the type I IFN that are produced by most cell types in response to virus infection, IFNγ is produced primarily by T-cells and NK cells in response to specific antigen or mitogens and plays a key role in T-cell activation and the establishment of the adaptive immune response [13,14]. A third IFN sub-family, the type III IFNs, has also been described recently [15,16]. In man, three individual type III IFNs have been identified, IFNλ1, IFNλ2, and IFNλ3, that are also known as IL-29, IL-28A, and IL-28B respectively. All three proteins are encoded by genes located on chromosome 19 [17] and bind to a specific cell-surface receptor comprising a unique IFNλR1 chain and the IL-10R2 chain, which is also shared, by the IL-10, IL-22, and IL-26 receptors [17]. Type III IFNs share a common signaling pathway with the type I IFNs, and activate the transcription of effector protein encoding genes [15,16,17]. In marked contrast, however, to type I IFNs, the type III IFN receptor is expressed only on certain cell types [15,16,17]. 

## 2. Type I Interferons

Type I IFNs exhibit antiviral, antiproliferative, and immunomodulatory activities and play a key role in the innate immune response to virus infection. Type I IFNs also enhance the expression of a number of apoptotic or pro-apoptotic genes including FAS, FAS ligand, pro-caspase 2 and 8, TRAIL, Bak, Bax, and the transcription factors IRF1 and IRF3 [18] IRF1 and IRF3 play a central role in the regulation of the interferon system and are themselves in turn induced by type I IFNs constituting a positive feed-back loop. Furthermore, IRF1 and IRF3 are also involved in the pro-apoptotic activity of the type I IFNs. Thus, the presence of infectious virus, inactivated viral particles, or viral component such as dsRNA, induce post-translational modification of IRF1 and IRF3 resulting in marked transcriptional activation of the apoptotic gene NOXA resulting in apoptosis of virus-infected cells [19], increased numbers of apoptotic vesicles, and enhanced presentation of viral peptides by IFN-activated dendritic cells (DCs). Thus, type I IFNs produced principally by plasmacytoid dendritic cells (pDCs) also play an important role in the establishment of the adaptive immune response by stimulating dendritic cell maturation through increased expression of co-stimulatory molecules including CD40, CD80, and CD86, and major histocompatability complex (MHC) antigens [20], and presentation of pathogen-derived antigens to naïve T-cells. Thus, following activation, DCs present virus-derived peptides on MHC class I antigens to CD8+ T leading to CD8+ T-cell activation [21]. Type I IFNs play an important role in this process, known as cross priming, independent of the CD40 ligand-CD40 interaction between DCs and CD+ T-cells [21,22,23]. IFNα can stimulate the priming and differentiation of naïve CD4+ lymphocytes potentiating a Th1 type response characterized by the production of IFNγ [24]. IFNα can also activate memory CD4+ T-cells when exposed to cognate antigen [25]. IFN can act directly on memory T-cells to increase their survival and indirectly through stimulation of IL-15 production by antigen presenting cells (APCs) that contributes to the survival of memory T cells [26,27]. Type I IFNs have been shown to induce B-lymphocytes to differentiate into antibody producing plasma cells [28] and to be necessary for the production of both specific and polyclonal IgG in response to virus infection and to stimulate isotype switching [29].

Recombinant analogues of IFNα2 and IFNβ1, and more recently pegylated forms of IFNα2, have found wide application for the treatment of chronic viral hepatitis and remitting relapsing multiple sclerosis (RRMS) respectively. Pegylated IFNs exhibit improved pharmacokinetics allowing less frequent injections without a loss of therapeutic activity [30]. Pegylated IFNs exhibit similar adverse events to those observed with the native molecule, however, even though plasma levels are more constant and peak-to-trough levels associated with frequent administration are reduced [31]. Pegylated IFNα2a (PEG-IFNα2a) or PEG-IFNα2b in combination with ribavirin is currently standard treatment for patients with chronic hepatitis C (HCV) and compensated liver disease [32]. IFNβ1a, and IFNβ1b are also widely used as first-line therapy for RRMS an autoimmune disease characterized by the presence of auto reactive T-cells in the central nervous system (CNS), demyelination, and axonal damage [33]. Type I IFNs are also used to treat neoplastic disease including hematological malignancies and solid tumors [34]. Thus, IFNα2 has been used to treat chronic hairy cell leukemia [34] chronic myelogenous leukemia [34], non-Hodgkin’s lymphoma in combination with chemo-therapy [34], and is used as an adjuvant to surgical restriction for the treatment of malignant melanoma [34,35]. 

Interferon induced adverse events are usually dose-dependant and to some extent a function of the IFN sub-species employed, the disease state, and the genetic makeup of the individual being treated. Adverse events include acute flu-like symptoms and sub-acute and chronic side effects such as myelosuppression, neuropsychiatric effects, and induction or exacerbation of underlying autoimmune disease.

### 2.1. Acute Adverse Events

Most patients treated with standard therapeutic doses of native or pegylated IFNα2 or IFNβ1 exhibit flu-like symptoms including fever, headache, chills, and myalgia within 1 to 3 hours of the initiation of IFN treatment. These symptoms decrease in intensity over time [36], and are believed to be due to both the release of the inflammatory cytokines IL-1, IL-6, and TNFα and induction of prostaglandin-E2 in the hypothalamus [37]. 

### 2.2. Sub-Acute and Chronic Side-Effects

#### 2.2.1. Myelosuppression

Neutropenia is one of the most common adverse events observed in patients with chronic hepatitis C treated with native or pegylated IFNα2a or IFNα2b [31,36]. Neutropenia can be sufficiently severe to necessitate dose-reduction in some 20% of patients and discontinuation of treatment in 2 to 6% of patients [31,36]. Anemia is also responsible for dose reduction in 25 to 28% of patients with chronic HCV infection treated with IFNα2 in combination with ribavirin [31,36]. Thrombocytopenia is also observed in approximately 6% of patients with chronic hepatitis C treated with PEG-IFNα2a or PEG-IFNα2b and often leads to dose reduction or discontinuation of treatment [38]. Hematological adverse events can be particularly severe in patients co-infected with HCV and HIV and treated with pegylated IFNα2 and ribavirin [39]. Sever hematological toxicity following IFN treatment is associated with cirrhosis, and low base-line body weight and hemoglobin levels [39]. The anti-proliferative effects of type I IFNs are thought to be responsible at least in part for the myleosuppression observed during IFN treatment. Thus, we have shown that human hematopoietic progenitor cells express functional type I IFN receptors [40] and IFNα has been shown to inhibit colony formation by human erythroid, myeloid, and megakaryocyte progenitor cells [41]. IFNα-induced leucopenia is also thought to result from an effect of IFNα on leukocyte trafficking between the peripheral circulation and lymphoid organs [36]. IFN-induced thrombocytopenia is thought to be due to an inhibition of megakaryocyte maturation and platelet production rather than an effect on megakaryocyte proliferation or endomitosis [42]. Thus, IFNα has been shown to inhibit the expression of GATA-1, p45^NF-E2^, and Mafg, transcription factors involved in late-stage megakaryopoiesis [42]. An association between polymorphisms in the genes encoding IFNAR1 and STAT2, and IFN-induced neutropenia has recently been reported in a Japanese population with chronic hepatitis C treated with type I IFN [43]. Similarly, IFN-induced thrombocytopenia was found to be associated with a polymorphism in the gene encoding IRF7 [43]. In contrast, these polymorphisms were not associated with a sustained virologic response to IFN therapy that was dependent upon independent factors including viral load, viral genotype and the degree of liver fibrosis on initiation of IFN treatment [43]. It will be of considerable interest to determine whether the same polymorphisms are also associated with IFN-induced hematological adverse events in populations of different ethnic origins.

#### 2.2.2. Hepatic toxicity

Elevated levels of liver transaminases are a common side effect observed during the first weeks of treatment of patients with IFNα. The effects are dose-dependant and resolve rapidly on cessation of IFN therapy. The mechanisms underlying these effects remain unclear [36].

#### 2.2.3. Autoimmune Disease Associated With Interferon Therapy

Apparent induction or exacerbation of preexisting autoimmune disease is a relatively frequent occurrence in patients treated with type I IFNs; cytokines that can affect both innate and adaptive immunity [44,45]. The genetic background of the individual as well as the characteristics of the disease treated will determine to a large extent the manifestations of autoimmune disease observed. Recent genome-wide association studies have identified alleles associated with susceptibility to a number of autoimmune diseases such as systemic lupus erythematosus (SLE) or Sjogren’s Syndrome (SS), some of which encode proteins involved in IFN induction or signaling pathways [46,47,48,49,50,51,52,53,54,55]. 

##### 2.2.3.1. Systemic lupus erythematosus (SLE), Cutaneous lupus erythematosus (CLE)

There are a number of reports of the induction or exacerbation of SLE, a disease that involves multiple internal organs, and CLE, which is limited to the skin, in patients treated with recombinant IFNα [45,56,57]. Patients with particular MHC class I and Class II haplotypes, female gender, and other risk factors, that predispose an individual to the development of autoimmune disease, are particularly susceptible to the development of IFN-induced SLE or CLE. The symptoms of IFN-induced SLE or CLE usually resolve after discontinuation of IFN treatment [45,56,57]. Although the mechanism(s) of IFN-induced SLE and CLE remain unclear, IFNα has been detected in the peripheral circulation of untreated SLE patients and a number of studies have shown an association between activation of the type I IFN pathway and disease activity [58,59]. Thus, the expression of a number of ISGs, often referred to as an “IFN signature”, is up regulated in peripheral blood mononuclear cells (PBMC) from SLE patients with active disease [58,60]. Furthermore, polymorphisms in the genes encoding IRF-5 and Tyk2, that play key roles in induction and the activity of IFNα respectively, have been identified as risk factors for the development of SLE [50,51]. A single nucleotide polymorphism (rs7574865) in the gene encoding the STAT4 protein, a transcription factor that is phosphorylated and activated following binding of IFNα to its receptor, has also been shown to be associated with risk of SLE in a population of European ancestry [61]. It has been suggested that auto-antibodies present in patients with SLE can form soluble immune complexes with RNA or DNA released by apoptotic or necrotic cells, and that these immune complexes are taken up by pDC leading to production of type I IFNs in a TLR-dependant [59]. It has also been reported that IRF-5, which is involved in induction of IFNα2 in the TLR 7/8 pathway, is expressed constitutively in pDCs [59]. Up-regulation of the IFNα inducible gene MxA is also observed in skin lesions of patients with CLE [56] that also contain large numbers of IFN-producing pDCs [62,63]. There is also evidence to suggest that IFNα is involved in the pathogenesis of the neuropsychiatric manifestations observed in some patients with SLE [64]. 

##### 2.2.3.2. Sjogren’s syndrome

Treatment of patients with standard therapeutic doses of type I IFNs has been reported to induce the symptoms of Sjogren’s syndrome [65,66]. Sjogren’s Syndrome is a relatively common systemic inflammatory disease characterized by production of antinuclear antibodies and a chronic cellular infiltration of the exocrine glands, in particular salivary and lachrymal glands leading to dry mouth (xerostomia) and dry eyes (keratoconjunctivitis sicca) respectively [67,68]. The cellular infiltrate consists principally of CD4+ T-cells, B-cells, and dendritic cells, including IFNα secreting pDCs [69]. It has been suggested that auto-antibodies present in patients with SS may form soluble immune complexes with U1 snRNA released by apoptotic or necrotic cells, and that these immune complexes are taken up by pDCs leading to production of IFNα via a mechanism involving TLR-7/8 [70]. In common with SLE, SS is characterized by enhanced expression of a number of ISGs and a type I IFN signature of gene expression is observed in both PBMCs and salivary glands [54,69,70,71]. Again in common with SLE, polymorphisms in the genes encoding IRF5 and STAT4, key transcription factors involved in IFN induction and action respectively, have been reported to be associated with SS in a number of different populations [52,53,54,72]. In contrast to these observations, both parenteral administration of moderate doses of IFNα, that is 1 to 2.3 × 10^6^ IU once to three times weekly respectively, or oral administration of low levels of IFNα (150 IU three times a day), have been reported to relieve the symptoms of xerostomia and xerophthalmia in patients with primary Sjogren’s Syndrome [73,74,75,76,77,78,79]. The mechanisms underlying these seemingly paradoxical effects remain to be elucidated.

##### 2.2.3.3. Dermatomyositis and polymyositis

The connective tissue diseases dermatomyositis and polymyositis, are characterized by underlying autoimmune abnormalities and up-regulation of numerous ISGs in various tissue of patients with active disease [80,81,82]. Both IFNα2 and IFNβ1 have been reported to induce or exacerbate dermatomyositis and polymyositis in some patients [82,83], and it have been suggested that as in the case of SLE and SS, RNA or DNA contained in soluble immune complexes taken up by pDCs interacts with TLRs in endosomal vesicles leading to local over-production of type I IFNs in skin and muscle [80,81,82]. 

##### 2.2.3.4. Systemic sclerosis

The rs7574865 polymorphism in the STAT4 gene and the rs2004640 polymorphism in the IRF5 gene are also associated with a predisposition to systemic sclerosis, an autoimmune connective tissue disorder characterized by fibrosis of multiple organs and a type I IFN signature [72,84,85]. Systemic sclerosis has been reported to occur following treatment with IFNβ1 [86]. IFNα2 has been reported to induce or exacerbate peripheral neuropathy in patients with HCV-associated mixed cryoglo-bulinemia [87]. There are also case reports of demyelinating neuropathies developing during IFNα2 treatment of patients with chronic hepatitis C without cryoglobulinemia [87]. Although it is well established that type I IFNs can enhance antibody production, up-regulate MHC class I antigen expression, and stimulate Th1 type immunity all of which may contribute to autoimmunity, the mechanism(s) by which IFNα exacerbates the autoimmune mechanisms underlying these diseases remains unclear [87].

##### 2.2.3.5. Rheumatoid arthritis (RA) and psoriatic arthritis

Exacerbation of the symptoms of RA or psoriatic arthritis has been reported in patients with chronic HCV infection or various malignancies treated with IFNα2, and in patients with RRMS treated with IFNβ1 [88,89]. Induction of new-onset RA is a relatively infrequent event in IFN-treated patients, and symptoms often resolve after cessation of treatment [88,90]. Symptoms may persist, however, in patients with a genetic predisposition [45,88]. In common with other autoimmune diseases such as SLE, single nucleotide polymorphisms in the gene encoding IRF5 have been shown to be associated with susceptibility to RA [91].

##### 2.2.3.6. Type I diabetes

IFNα has been reported to induce symptoms of insulin-dependent type I diabetes in patients with chronic hepatitis C infection; for the most part in individuals with pre-existing autoimmunity and/or with a genetic predisposition [45,92]. As in the case of other autoimmune diseases the action of IFNα may be related to increased expression of MHC class I antigens, enhanced expression of co-stimulatory molecules, and activation of DCs as well as stimulation of immunoglobulin synthesis and increased autoantibody production, and an enhanced T-cell response leading to the destruction of pancreatic β-cells. There is also convincing evidence from both clinical studies and animal models to suggest that IFNα is indeed involved in the pathogenesis of type I diabetes [92,93,94,95].

##### 2.2.3.7. Autoimmune hepatitis

Some cases of autoimmune hepatitis have been reported in patients with chronic HCV infection, or malignancies treated with IFNα and in patients with RRMS treated with IFNβ1 [96,97,98,99,100,101,102]. Autoimmune hepatitis is a rare but sever adverse event in IFN-treated patients that is often life threatening and is associated with certain HLA haplotypes [96,97,98,99,100,101,102].

#### 2.2.4. Neuropsychiatric effects associated with Interferon therapy

Treatment of patients with chronic HCV infection with native or pegylated IFNα is associated with a number of neuropsychiatric side-effects including fatigue, depression, cognitive disturbances and in some cases suicidal tendencies [103]. IFNα can also cause cognitive dysfunction that dissipates on cessation of IFN treatment [103,104]. Although some of these effects may be due in part to the consequences of somatic toxicity they may also be due to effects of IFNs on neurons, serotonergic activity, neuroendocrine cytokine secretion, tryptophan metabolism and neurotransmitters. IFNα has to affect the hypothalamic-pituitary-adrenal system by activating corticotrophin-releasing factor [64,102,103,104,105,106,107,108]. Several studies have also shown that IFNα can cause changes in the electrophysiological activity of the brain [64,102,103,104,105,106,107,108]. Increased circulating levels of IL6 observed during IFNα therapy are associated with symptoms of depression and high levels of IL6 prior to treatment are predictive of depression during IFN treatment [109,110]. Functional polymorphisms in the promoter of the IL6 gene and in the promoter region of the serotonin transporter (5-HTT) gene [111] have been shown to be associated with a lower risk of symptoms of depression during IFNα treatment. Neither polymorphism, however, affects the development of fatigue that occurs during IFNα therapy [111]. 

Systemic administration of type I IFNs can result in activation of IFN receptors present on essentially all types of nucleated cells, including neurons [104,112]. Although type I IFNs do not cross the blood-brain barrier under physiological conditions, permeability may be altered during virus infection, or during various inflammatory disease. IFNs can also active a number of cell types present in the central nervous system (CNS) such as neurons, astrocytes, microglia, as well as cells such as macrophages or T-lymphocytes that may enter the CNS in patients with chronic viral hepatitis, or inflammatory demyelinating disease [104,113]. Production of IFNα has also been implicated in the development of the neuropsychiatric effects, including psychosis, observed in some patients with SLE.

#### 2.2.5. Neuromuscular disorders associated with Interferon therapy

Peripheral neuropathy is a rare adverse event observed in some patients with chronic hepatitis C treated with native or pegylated IFNα [87,113]. In patients with chronic myeloid leukemia or in HCV-infected patients with mixed cryoglobulinemia and neuropathy, IFNα has also been reported to either exacerbate pre-existing disease or induce new onset neuropathy [87,113]. Some cases of the development or exacerbation of pre-existing autoimmune demyelinating neuropathies have also been reported in patients with chronic hepatitis C treated with IFNα [87]. There are isolated case reports of axonal neuropathies associated with IFNα therapy although the pathogenesis of axonal injury by IFNα remains unknown [87]. IFNα treatment has also been reported to exacerbate myasthenia gravis in some HCV-infected patients, or in patients with various malignancies treated with IFNα [87,114,115]. There are also isolated reports of the appearance of neuromotor abnormalities such as dystonia, chorea, myorhythmia, and Parkinson’s disease in patients treated with native or pegylated IFNα [116,117]. There is evidence from animal models to suggest that IFNα can exert an inhibitory effect on nigrostriatal dopaminergic transmission which may account at least in part for some of the Parkinson disease like symptoms observed in some patients [116,117,118].

#### 2.2.6. Thyroid dysfunction associated with Interferon Therapy

Thyroid dysfunction including hypothyroidism and less frequently hyperthyroidism, is a relatively common side-effect of IFN treatment that occurs in approximately 6% of IFN treated patients, although the incidence varies considerably from one study to another [119]. IFN-induced thyroid dysfunction is often sub-clinical and resolves spontaneously in a majority of patients [119]. Both IFNα and IFNβ therapy are associated with the development of thyroid dysfunction and although differences in the dose and frequency of IFN treatment may well account in part for this variability, the incidence of thyroid dysfunction is higher in IFN-treated patients with pre-existing thyroid autoimmunity irrespective of the disease being treated [119]. Although the mechanisms leading to IFN-induced thyroid dysfunction remain unclear, immune modulation by type I IFNs may well exacerbate underlying immune dysfunction in patients with pre-existing thyroid autoimmunity. Direct effects of type I IFNs on thyroid cells resulting in an inhibition of iodine uptake and thyroxin secretion may also contribute to the thyroid dysfunction observed in IFN treated patients [119,120].

#### 2.2.7. Sarcoidosis associated with Interferon Therapy

Sarcoidosis is a chronic granulomatous disorder of unknown etiology that can affect numerous organs including the lungs, skin, eyes, and liver. Sarcoidosis involving most frequently the lungs and skin has been reported in a number of patients with chronic hepatitis C or various malignancies treated with native or pegylated IFNα [121,122] and less frequently in patients treated with IFNβ1. Symptoms tend to regress spontaneously either during treatment or after cession of IFN treatment [121,122,123,124,125,126,127]. It has been suggested that the induction or exacerbation of Sarcoidosis during IFNα therapy may be to IFNα stimulation of IFNγ secreting activated T-lymphocytes [45].

## 3. Interferon Lambda

Recent independent genome-wide association studies have identified single nucleotide polymorphisms in the region of the IFNλ3 (IL28B) gene that are associated with the response of patients with chronic HCV infection to treatment with PEG-IFNα2/ribavirin [128,129,130]. In common with type I IFNs, IFNλ3 exhibits antiviral activity against HCV and small quantities of IFNλ3 can potentiate the antiviral activity of IFNα2 and *vice versa* [131]. Although type III IFNs share a common signaling pathway with the type I IFNs, and activate the transcription of a similar set of interferon sensitive genes (ISGs) encoding IFN effector proteins [131], difference in the kinetics of ISG activation by IFNλ1 and IFNα2 have been reported [131]. Furthermore, initial clinical studies with pegylated IFNλ1 suggest that IFNλ1 exhibited anti-HCV activity in the absence of the flu-like symptoms or hematological side effects associated with treatment with type I IFNs [132]. The most common side effects observed in patients with chronic HCV infection treated with PEG-IFNλ1 were myalgia and fatigue [132]. These results may well be related to the more restricted distribution of the IFNλ receptor expression. Thus, the IFNλ receptor does not appear to be expressed on monocytes or lymphocytes [133] and the IL-10R2 chain of the IFN lambda receptor is expressed in very low levels in certain compartments of the brain [134]. Recent results have shown that IFNλ has a relatively modest activity in the brain suggesting that the therapeutic use of IFNλ may be associated with less neurological side effects [132]. IFN lambda in association with ribavirin and/or IFNα2 holds considerable promise for the treatment of chronic viral hepatitis with the prospect of less side effects than current treatment options.

## 4. Interferon γ

IFNγ production is critical for the establishment of the adaptive immune response, T-helper Th1 cell response, and protective immunity against viruses and intracellular bacteria [14]. IFNγ is produced principally by NK cells and activated T-cells in response to specific antigen, mitogens, IL-2, IL-12, and IL-18 upon subsequent stimulation with IL-12 [135]. IFNγ plays an important role in the activation and differentiation of NK cells, B-cell, T-cells, and macrophages [14]. IFNγ also enhances expression of MHC class II antigens on antigen presenting cells (APCs), increases antigen presentation to helper T-cells [135], and plays a key role in CD8+ T-cell activation [14]. 

Despite its key role in the establishment of the adaptive immune response, IFNγ has not found wide application in the clinic [135,136]. IFNγ, is used principally for the treatment of chronic granulomatous disease (CDG), a rare congenital disorder characterized by a lack of superoxide and hydrogen peroxide production by macrophages and susceptibility to bacterial and fungal infections [136]. IFNγ is also used for the treatment of congenital osteopetrosis a rare pediatric disorder characterized by a defect in osteoclast function [137]. IFNγ alone has been shown to be without benefit in the treatment of chronic hepatitis B or chronic hepatitis C and to be poorly tolerated when combined with IFNα treatment [136]. Similarly, IFNγ is not used for the treatment of multiple sclerosis and although IFNγ has shown some benefit in the treatment of ovarian cancer it is has had not been widely used to treat neoplastic disease [14,136].

In common with type I IFNs, treatment with IFNγ is generally well tolerated and the most common adverse events observed are “flu-like” symptoms including fever, chills, fatigue, headache, and myalgia similar to those observed in patients treated with type I IFNs [136,138]. IFNγtreatment is also associated with reversible neutropenia and thrombocytopenia that may be severe in some patients, and marked increases in hepatic transaminases [14,136,138]. IFNγ receptors are ubiquitous and a number of the genes activated by IFNγ are also activated by type I IFNs. This may well account for a spectrum of toxicities similar to that associated with type I IFNs including “flu-like” symptoms, neutropenia, thrombocytopenia, and increased levels of hepatic transaminases. It is difficult to determine, however, whether other adverse effects observed in patients treated with type I IFNs, such as neuropsychiatric effects or induction or exacerbation of preexisting autoimmune disease would also be observed in patients treated with IFNγ due to the relatively limited clinical application of IFNγ therapy. Development of symptoms of SLE has been reported, however, in patients with RA treated with recombinant IFNγ [139,140]. IFNγ has also been implicated in the pathogenesis of sarcoidosis, an autoimmune disease characterized by a Th1 dominant cytokine profile, with high levels of IL-12, IL-18, IFNγ, and IP-10, in the bronchoaleveolar lavage [141,142,143,144,145], it remains to be established whether treatment with recombinant IFNγ would induce or exacerbate underlying sarcoidosis as has been reported in patients treated with IFNα [121,122,123,124,125,126,127] and in patients treated with IFNα combined with IFNγ [146]. 

## 5. Immunogenicity

Repeated administration of recombinant IFNα2 or IFNβ1, can result in a break in immune tolerance to self-antigens in some patients resulting in the production of anti-IFN antibodies. These antibodies may bind to the IFN molecule in such a manner that they are largely without effect, or they may alter IFN pharmacokinetics, or in certain cases neutralize the activity of the IFN by binding to an epitope that prevents IFN from binding to its cell-surface receptor on target cells [147,148]. The results of a number of clinical studies have shown that the development of anti-IFNα antibodies occurs frequently in patients with chronic hepatitis C or neoplastic disease, treated with either IFNα2a or IFNα2b [149]. The incidence of anti-IFN antibody production is highly variable, however, and has been reported to vary from 7 to 60% [149]. A lack of assay standardization and differences in treatment regiments undoubtedly accounts in part for the apparent variation in the incidence of anti-IFN antibodies in patients treated with recombinant IFNα. In most of these studies, however, the incidence of neutralizing antibodies was low [149]. Pegylation increases the plasma half-life of IFNα permitting once-weekly dosing with improved efficacy compared with the native molecule. Pegylation is often considered to reduce the immunogenicity of recombinant proteins by PEG-induced steric hindrance and reduced immune recognition [31,150,151]. It has been reported, however, that some 8% of patients with chronic hepatitis C infection who failed to respond to therapy with pegylated IFNα2 and ribavirin, had circulating neutralizing anti-IFNα antibodies while none of the patients who cleared HCV following IFN therapy had detectable levels of anti-IFNαNAbs [152]. 

The incidence of anti-IFN antibodies appears to be higher in patients with RRMS treated with recombinant IFNβ1 than in patients with chronic hepatitis C treated with either native or pegylated IFNα2 [149,153,154]. Direct comparisons are difficult however, due to different dosing regimens, differences in the duration of treatment, and differences in the genetic background of the individuals treated. In addition, differences in formulation can influence protein aggregation that can in turn affect immunogenicity [155]. The use of different assays and reference preparations can also influence the results obtained. It remains to be determined whether the apparent differences in the immunogenicity of IFNα or IFNβ subspecies are due to intrinsic differences in the primary structure or glycosylation of the molecules. Similarly, although differences in the immunogenicity of IFNβ1a and IFNβ1b have been reported [156]. Thus, analysis of 846 patients with RRMS treated with IFNβ showed that 88% of patients receiving IFNβ1b, 45% of patients treated with IFNβ1a IM, and 56% treated with IFNβ1a SC developed antibodies against IFNβ at some point during 1 to 4 years of treatment, and that neutralizing antibodies were detected in 37%, 12%, and 51% of these patients, respectively [156]. It remains to be determined whether the apparent differences in the immunogenicity are due differences in primary structure or to the use of different expression systems and manufacturing procedures [155]. The results of a number of studies have shown that polyclonal antibodies produced in patients treated with IFNα2a or IFNα2b cross-neutralize both proteins as well as pegylated derivatives of either protein or native IFNα1 [152,157]. Similarly, sera from patients with RRMS treated with IFNβ1a (Avonex or Rebif) have been shown to cross-neutralize IFNβ1b (Betaferon). The lack of cross-neutralization of IFNβ by anti IFNα NABs and *vice versa*, undoubtedly accounts for the apparent lack of untoward effects associated with the presence of anti-IFN NABs with the exception of relatively mild infusion/injection reactions. This is in marked contrast to the sever autoimmune reactions associated with the presence of auto-antibodies to non-redundant essential proteins such as erythropoietin (EPO) or megakaryocyte growth and development factor (MGDF) that can result in pure red cell aplasia and sever thrombocytopenia respectively [158,159]. The results of a number of clinical studies have shown, however, that the development of anti-IFN NABs is associated with both reduced bioavailability and a reduced clinical response in patients treated with either IFNα2 or IFNβ1 [149,152,153,154]. Thus, in RRMS patients treated with IFNβ the development of anti-IFN NABs is associated with both reduced pharmacodynamics and reduced induction of IFNβ responsive gene products [147], and a reduced clinical response, determined by either magnetic resonance imaging (MRI) or disease progression [154,160,161].

There is limited data available on the immunogenicity of IFNγ. It has been suggested that recombinant IFNγ is not immunogenic, based on a study 334 patients most of whom were treated for short periods with IFNγ in a series of Phase I and II clinical trials [162]. One patient, however, did develop transient non-neutralizing anti-IFNγ antibodies after cessation of a three months intravenous treatment regimen [162]. Furthermore, there are several reports of the presence of IFNγ autoantibodies in patients presenting with recurrent *Mycobacterium* infections [163,164,165,166,167,168].

## 6. Conclusions

Since their introduction in the early eighties recombinant interferons have found wide application for the treatment of chronic viral hepatitis, neoplastic disease, and remitting relapsing multiple sclerosis. A large body of information is now available on their efficacy, safety, tolerability, and immunogenicity. For the most part interferons are well tolerated, with the most common adverse events observed being “flu-like” symptoms that resolve rapidly after initial treatment. Prolonged treatment is associated, however, with more serious adverse events including leucopenia, thrombocytopenia, and neuropsychiatric effects that may necessitate dose reduction or even cessation of IFN treatment in some patients. Polymorphisms in type I IFN signaling molecules are associated with IFN-induced neutropenia, thrombocytopenia, and neuropsychiatric effects, raising the possibility that patients most likely to well tolerate IFN therapy can be identified. Similarly, polymorphisms in IFN signaling molecules are associated with IFN-induced autoimmune disease again suggesting that individuals may be stratified according to the risk of developing symptoms of autoimmune disease during IFN therapy. Repeated administration of recombinant IFNs can cause in a break in immune tolerance in some patients resulting in the production of a polyclonal antibody response that can adversely affect pharmacokinetics, and clinical response. Recent studies have shown that the immunogenicity of IFNβ1 can be reduced by changes in formulation [169] opening the way for less immunogenic and better-tolerated IFNs. The recently discovered type III IFNs that are currently in clinical development, bind to a receptor restricted to certain cell types suggesting that IFNλ therapy may be associated with a reduced spectrum of toxicities relative to type I or type II IFNs, that bind to receptors present on essentially all nucleated cells. The results of initial clinical trails with PEG-IFNλ1 suggest that the apparent absence of flu-like symptoms or hematological side effects may indeed be related to the relatively restricted pattern of IFNλ receptor expression. The advent of less immunogenic and better tolerated IFNs together with individualized therapy suggests that IFNs will continue to play an important role in the future treatment of human disease.
